# Supplementary material for: Vascular endothelial-derived SPARCL1 exacerbates viral pneumonia through pro-inflammatory macrophage activation
Source: Nat Commun. 2024 May 18;15:4235. doi: 10.1038/s41467-024-48589-3 (PMC11102455; doi:10.1038/s41467-024-48589-3)
Supplement: Supplementary file 1 — Supplementary Information [file 41467_2024_48589_MOESM1_ESM.pdf]

Supplementary Information

Vascular Endothelial-derived SPARCL1 Exacerbates Viral Pneumonia Through Pro-Inflammatory Macrophage Activation

Gan Zhao* et al… Andrew E. Vaughan*

*Co-corresponding authors

Supplementary Figures 1-16
Supplementary Tables 1-2
References

Supplementary Fig.1

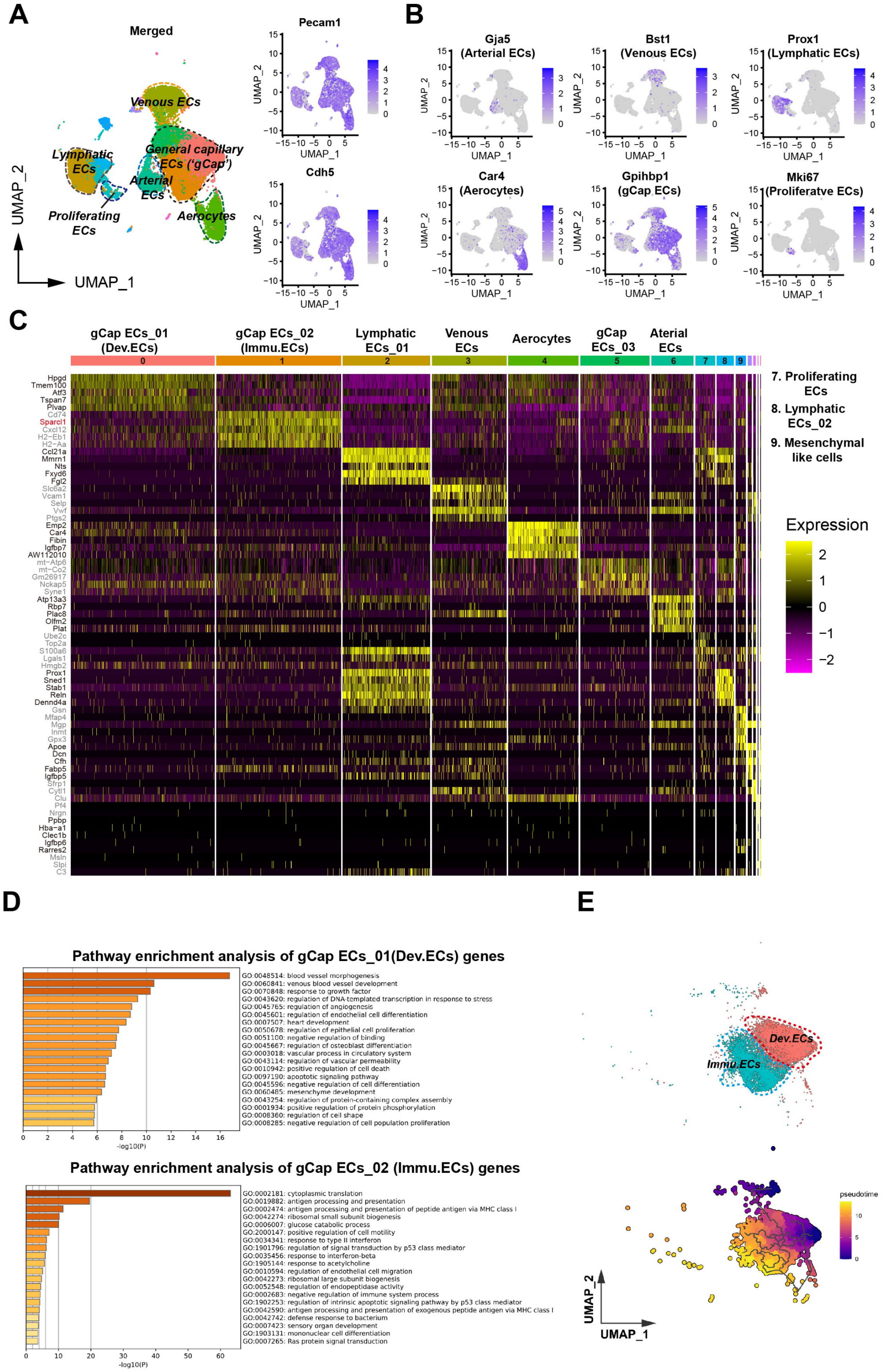

Supplementary Fig.1 Single-cell transcriptomics reveals heterogeneous changes in pulmonary vascular endothelial cells after influenza injury

A. UMAP plots showed the signature EC genes *Pecam1*(CD31), *Cdh5* and the major EC clusters annotated for mouse lungs.

B. Feature plots for identified EC cluster signature genes[1, 2].

C. Heatmap of the most differentially expressed genes (Top 5) of lung ECs in all clusters. The color bars indicate gene expression level in log2 scale.

D. The GO terms of differentially expressed genes in Dev.ECs and Immu.ECs were carried out by Metascape[3].

E. Pseudotime analysis of 2 gCap clusters of interest.

Supplementary Fig.2

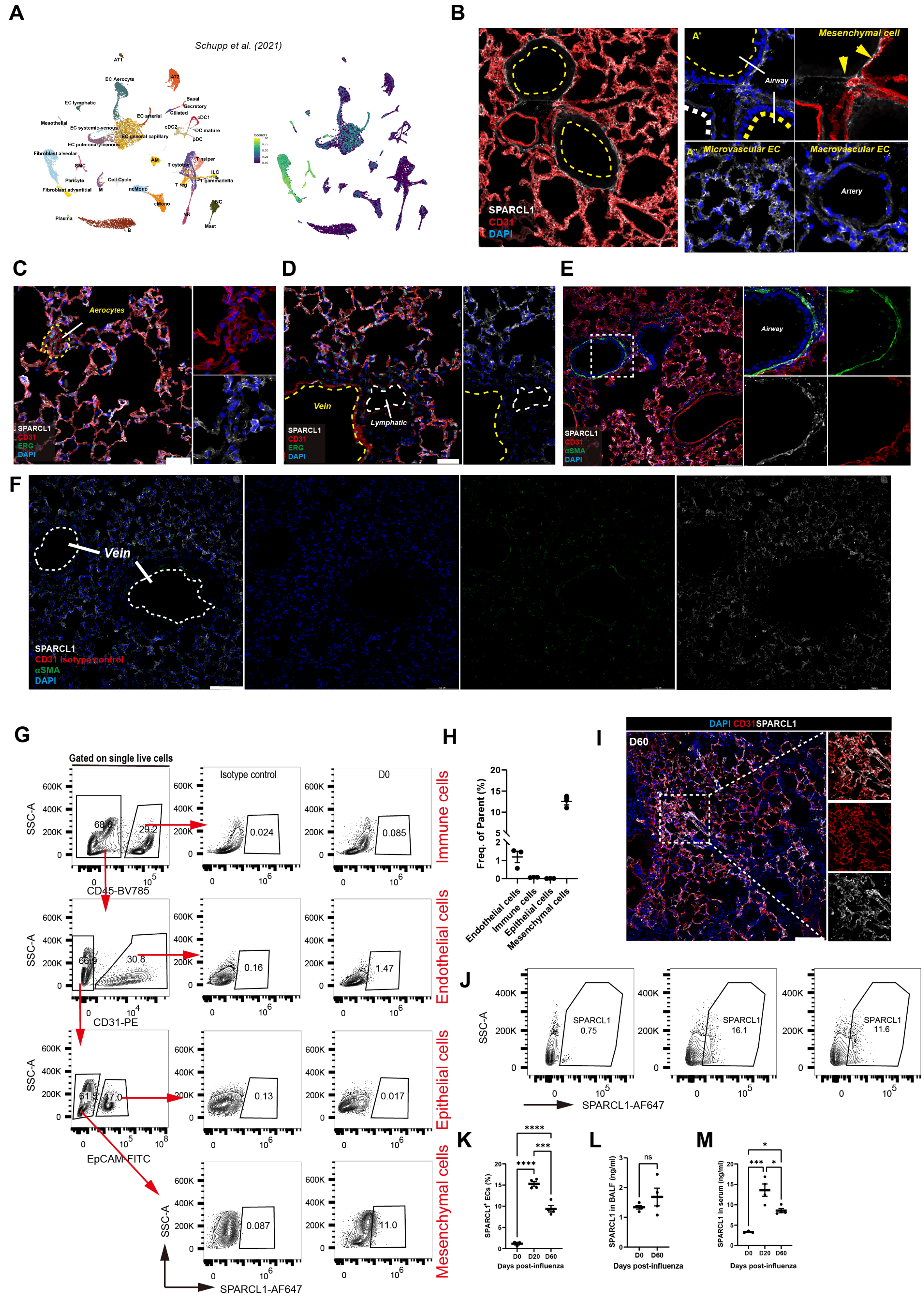

Supplementary Fig.2 In mouse lung, SPARCL1 is mainly expressed in capillary ECs and stromal cells

A. From a publicly available single-cell transcriptome dataset[4] (Lung Endothelial Cell Atlas: http://www.lungendothelialcellatlas.com/) of healthy mouse lungs, *Sparcl1* is revealed to be predominantly expressed in endothelial and stromal cells.

B. Mouse lung tissues (uninjured) were stained with SPARCL1, ERG and CD31/PECAM1, demonstrating SPARCL1 expressed in stromal/mesenchymal cells (**A'**), lung capillary ECs (**A''**), scale bar: 50 μm.

C. SPARCL1 is low / absent in aerocyte ECs (aCap ECs), scale bar: 50 μm.

D. SPARCL1 is low / absent in large vessel ECs, including arterial (**A''**) and venous ECs and lymphatic ECs, scale bar: 50 μm.

E. SPARCL1 expressed in stromal cells (α-SMA^+) lining airways.

F. Mouse lung tissues (uninjured) were stained with SPARCL1, α-SMA and CD31/PECAM1 Isotype control antibody, scale bar: 100 μm.

G-H. Intracellular flow cytometry analysis of SPARCL1 expression in immune cells ($CD45^+$), endothelial cells ($CD31^+$), epithelial cells ($EpCAM^+$), and stromal or fibroblast cells ($CD45^-CD31^-EpCAM^-$) from the lungs of mice without influenza infection (D0). (**G**), typical gating strategy; (**H**), Quantitative distribution of SPARCL1 expression in different cell populations in the lungs of D0 mice, gated as shown in (G).

I. Representative immunostaining of SPARCL1 in endothelial cells (CD31) on day 60 (D60) after influenza infection lung tissues. Scale bar, 100 μm.

J-K. Intracellular flow cytometry analysis of SPARCL1 expression in endothelial cells ($CD31^+$), from the lungs of mice on day 20 (D20) and D60 after influenza infection. (K) is quantification of all samples gated as shown in (J).

L-M. The concentration of SPARCL1 in bronchoalveolar lavage fluid (BALF) and serum was measured by ELISA at 0 (uninjured), 20, and 60 days after influenza infection, each dot represents one mouse, n = 3-4 mice per group, independent biological replicates.

Data in (**K**) and (**M**) are presented as means ± SEM, calculated using one-way analysis of variance (ANOVA), followed by Dunnett's multiple comparison test. Data in (**L**) is presented as means ± SEM, calculated using unpaired two-tailed t test. *P < 0.05, **P < 0.01, ***P < 0.001 and ****P < 0.0001. Source data are provided as a Source Data file.

Supplementary Fig.3

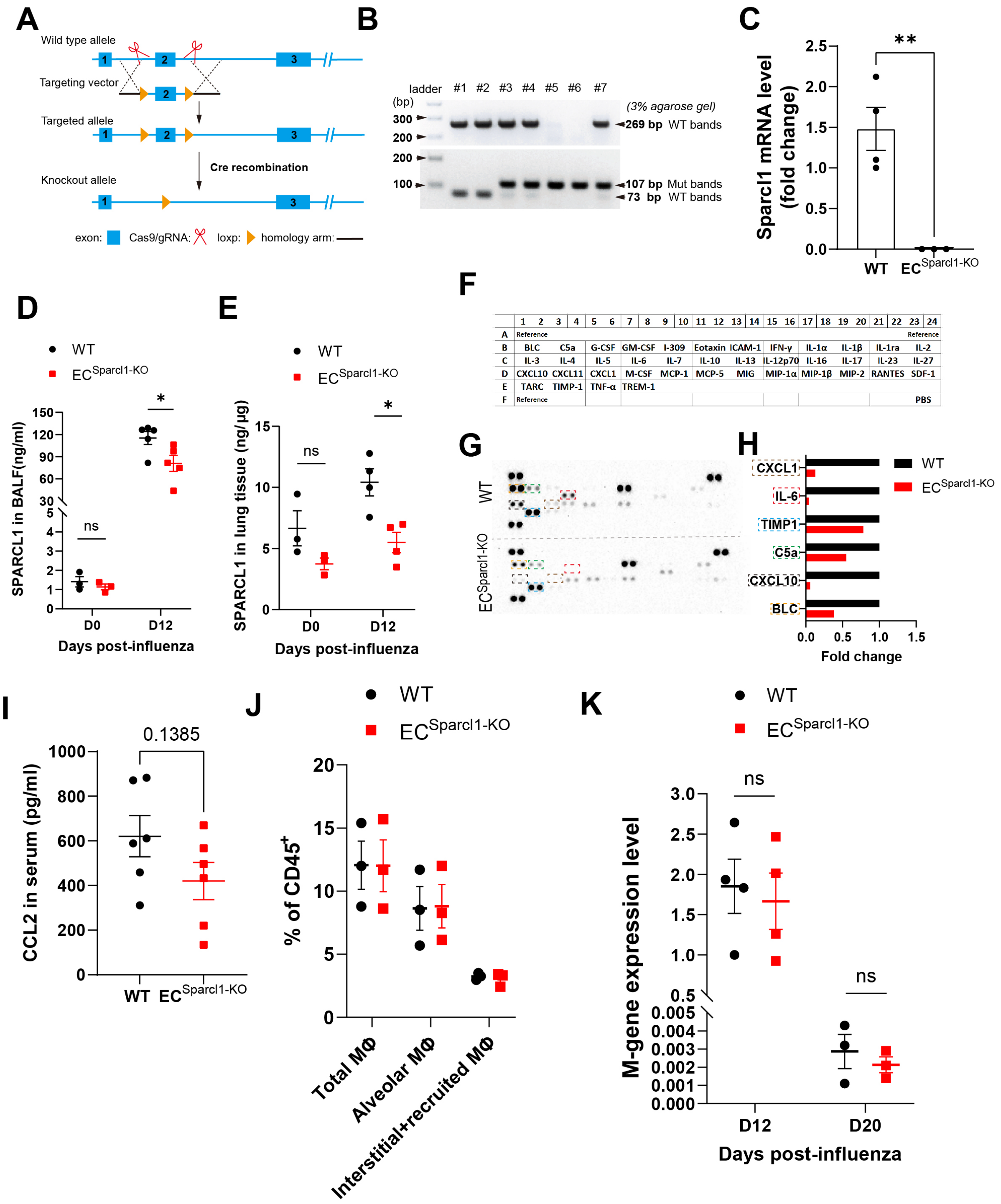

Supplementary Fig.3 Endothelial *Sparcl1* deficiency attenuates the accumulation of pro-inflammatory cytokines in the lungs

A. Schematic of the strategy used to generate Sparcl1$^{loxP/+}$ mice using CRISPR/Cas9 technology.
B. DNA gel image of Sparcl1^{flox} mouse genotyping. Lanes 1 and 2 represent wild-type mice with PCR product at 269 bp. Lanes 3, 4 and 7 represent heterozygous mice with product at 269 bp (wt) and product at 113 bp (Primers designed upstream and downstream of the loxp site). A light band at 79 bp from amplification of the WT locus can be observed as well in heterozygous mice but additional primers genotyping for WT allele (upper) is used to confirm. Lanes 5 and 6 represent homozygous mice, where LoxP sites were inserted at both alleles (single 119-bp PCR product).
C. qPCR analysis of *Sparcl1* in isolated lung ECs (CD45^{-}CD31^{+}) sorted from $EC^{Sparcl1\text{-}KO}$ (VECad^{CreERT2}; Sparcl1$^{flox/flox}$) or WT (Sparcl1$^{flox/flox}$) mice 2 weeks after 5 doses of tamoxifen. n = 3-4 mice per group, independent biological replicates.
D-E. ELISA assay to detect the concentration of SPARCL1 in BALF (**D**) and serum (**E**) from WT (wild-type) and $EC^{Sparcl1\text{-}KO}$ mice at day 0 and 12 after infection. each dot represents one mouse, n =3-5 mice per group, independent biological replicates.
F-H. Cytokines array. (**F**), Representative images for cytokine array panels. (**G**), The cytokine and chemokine levels in the BALF from WT and $EC^{Sparcl1\text{-}KO}$ mice at day 12 after infection analyzed by a Proteome Profiler Mouse Cytokine Arrays. (**H**), Results obtained from cytokine/chemokine arrays are visualized in the bar graph, values are presented as fold change over the WT group. BALF samples were pooled from 4 mice per group, independent biological replicates.
I. CCL-2 concentration in serum from WT (wild-type) and $EC^{Sparcl1\text{-}KO}$ mice at day 12 after infection. n = 6 mice per group, independent biological replicates.
J. Quantification of total lung macrophages (CD64^{+}F4/80^{+}), alveolar macrophages (CD64^{+}F4/80^{+}SiglecF^{+}) and interstitial and recruited macrophages (CD64^{+}F4/80^{+}SiglecF^{-}) under homeostasis in WT and $EC^{Sparcl1\text{-}KO}$ mice. n = 3 mice per group, independent biological replicates, gated as shown in Supplementary Figure.11A.
K. Expression of the influenza M-gene in mouse lungs on day 12 (n =4 independent biological replicates/group) and 20 (n =3 independent biological replicates/group) post infection. Normalized to expression on D12.
Data in (**C**), (**D**), (**E**), (**I**), (**J**) and (**K**) are presented as means ± SEM, calculated using unpaired two-tailed t test. *P < 0.05, **P < 0.01, ns: not significant, P>0.05. Source data are provided as a Source Data file.

Supplementary Fig.4

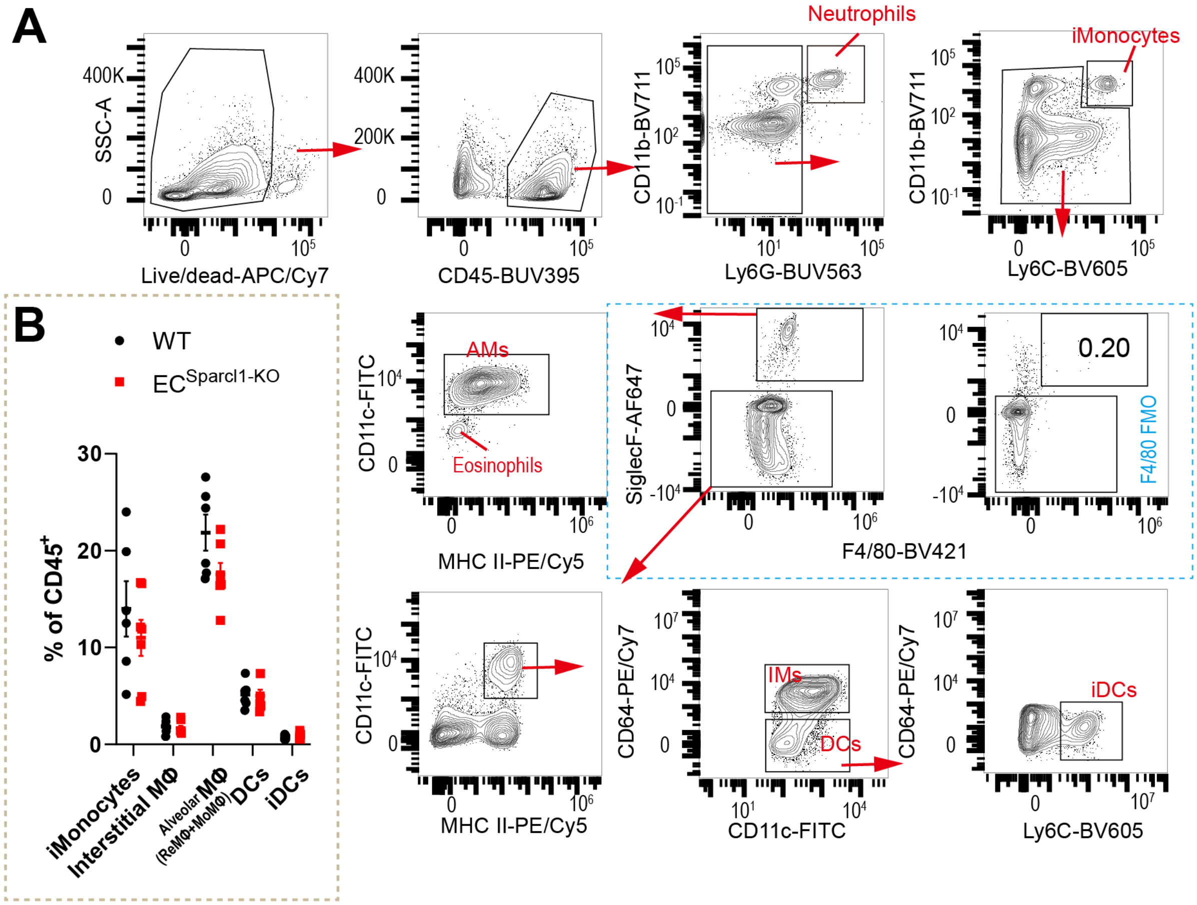

Supplementary Fig.4 Endothelial loss of *Sparcl1* reduced the recruitment of alveolar macrophages

A. Gating strategy for lung myeloids, including neutrophils ($CD11b^+Ly6G^+$), inflammatory monocytes (iMonocytes, $CD11b^+Ly6C^+$), resident and monocyte-derived alveolar macrophages (AMs, $F4/80^+SiglecF^+CD11c^+$), interstitial macrophages (IMs, $SiglecF^-F4/80^+CD11c^+MHC\text{-}II^+CD64^+$), dendritic cells (DCs, $SiglecF^-F4/80^+CD11c^+MHC\text{-}II^+CD64^-$) and inflammatory DCs (iDCs, $Ly6C^+$ DCs).

B. The effects of endothelial *Sparcl1* deficiency on the quantity of lung myeloid populations on day 12 after influenza infection. WT: n = 6 mice, independent biological replicates, $EC^{Sparcl1\text{-}KO}$: n = 5 mice, independent biological replicates. Data are presented as means ± SEM and calculated using unpaired two-tailed t test, gated as shown in (A). Source data are provided as a Source Data file.

Supplementary Fig.5

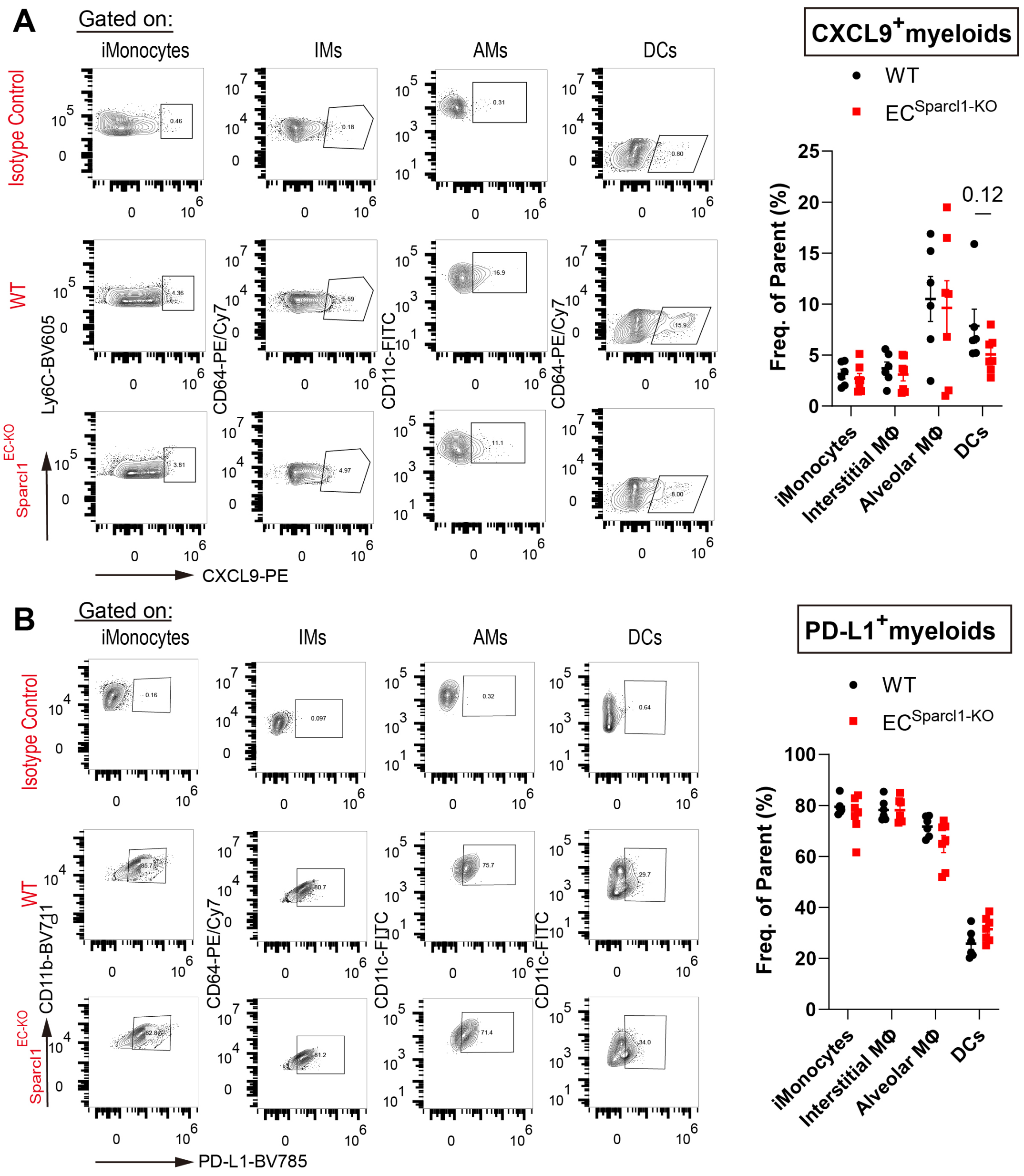

Supplementary Fig.5 The effect of endothelial loss of Sparcl1 on myeloid cells expressing CXCL-9 or PD-L1 on day 12 post-infection. Quantification of CXCL9(**A**) and PD-L1(**B**)-expressing myeloids, including inflammatory monocytes (iMonocytes), alveolar macrophages (AMs), interstitial macrophages (IMs) and dendritic cells (DCs), gated as shown in Supplementary Figure.4A. WT: n = 6 mice, $EC^{Sparcl1\text{-}KO}$: n = 7 mice, independent biological replicates. Data are presented as means ± SEM and calculated using unpaired two-tailed t test. *P < 0.05, ns, no significance, P >0.05. Source data are provided as a Source Data file.

Supplementary Fig.6

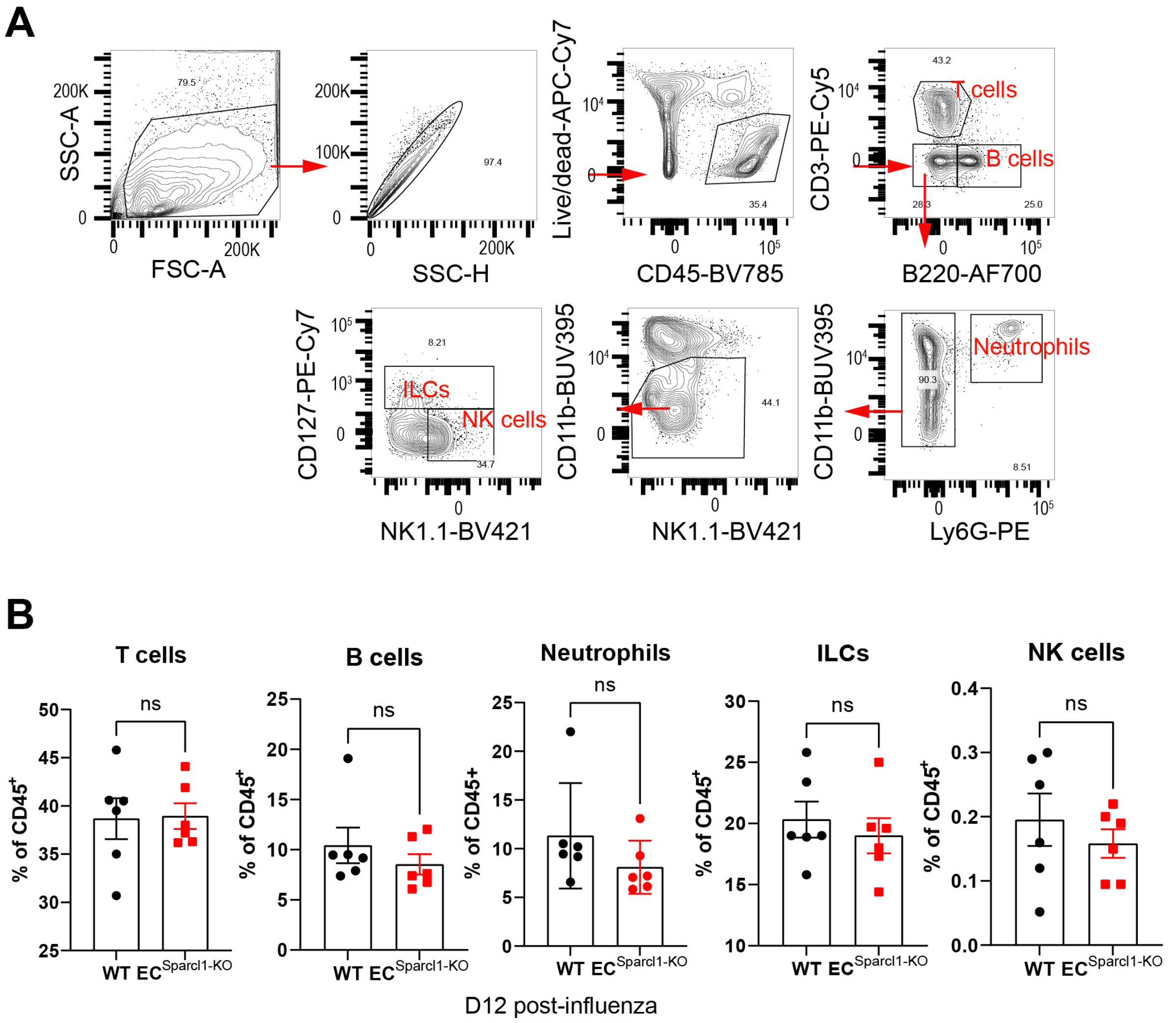

Supplementary Fig.6 Endothelial loss of *Sparcl1* does not impact the frequency of T, B, neutrophils, NK and innate lymphoid cells during influenza-induced lung injury

A. Representative gating scheme for flow cytometry analysis of T, B, neutrophils, NK, and innate lymphoid (ILC) cells in mouse lungs on day 12 after influenza infection.

B. Quantification of the proportion of T cells (CD3^{+}B220^{-}), B cells (B220^{+}CD3^{-}), neutrophils (CD11b^{+}Ly6G^{+}), NK cells (B220^{-}CD3^{-}Ly6G^{-}CD11b$^{low/-}$CD127^{-}NK1.1^{+}) and ILCs (B220^{-}CD3^{-}Ly6G^{-}CD11b$^{low/-}$CD127^{+}) CD45^{+} live cells at day 12 after influenza infection in WT and $EC^{Sparcl1\text{-}KO}$ mice, n = 6 mice per group, independent biological replicates, gated as shown in (A). Data are presented as means ± SEM and calculated using unpaired two-tailed t test. *P < 0.05, ns, no significance, P >0.05. Source data are provided as a Source Data file.

Supplementary Fig.7

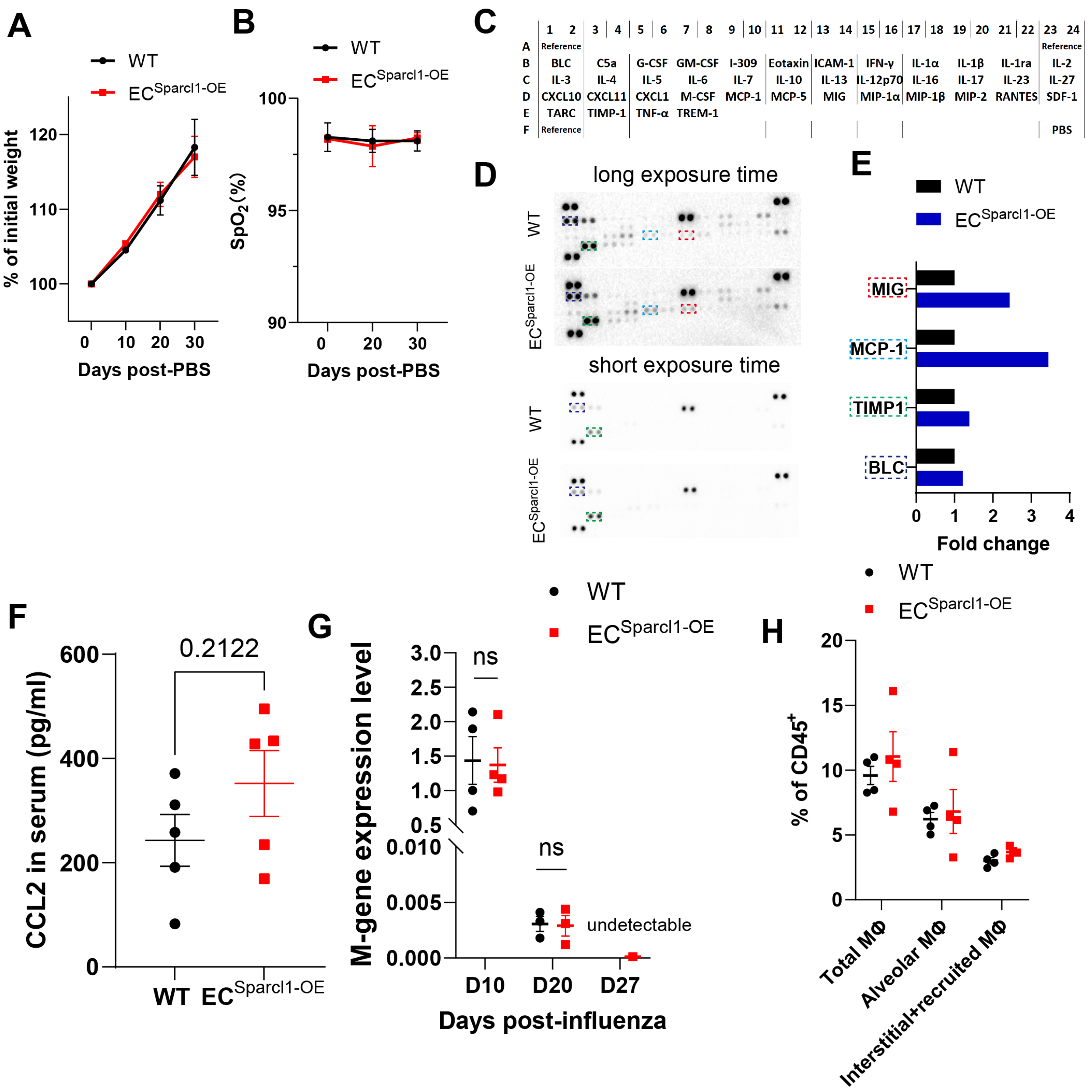

Supplementary Fig.7 Endothelial overexpression of *Sparcl1* increased pro-inflammatory cytokine levels in the lung during injury

A-B. Time course of changes in body weight (**A**) and capillary oxygen saturation (**B**) in WT and $EC^{Sparcl1\text{-}OE}$ mice after PBS administration, n = 3 to 4 mice per group, independent biological replicates.

C-E. Cytokines array. (**C**), Representative images for cytokine array panels. (**D**), The cytokine and chemokine levels in the BALF from WT and $EC^{Sparcl1\text{-}OE}$ mice at day 20 after infection analyzed by a Proteome Profiler Mouse Cytokine Arrays. (**E**), Results obtained from cytokine/chemokine arrays are visualized in the bar graph, values are presented as foldchange over the WT group. BALF samples were mixed by 4 mice per group, independent biological replicates.

F. CCL-2 concentration in serum from WT (wild-type) and $EC^{Sparcl1\text{-}KO}$ mice at day 12 after infection. n = 6 mice per group, independent biological replicates.

G. Expression of the influenza *M-gene* in mouse lungs on day 10 (n =4 independent biological replicates per group), 20 (n = 3 independent biological replicates per group mice) and 27(n =4 independent biological replicates per group) post infection. Normalized to expression on D10.

H. Quantification of total lung macrophages ($CD64^{+}F4/80^{+}$), alveolar macrophages ($CD64^{+}F4/80^{+}SiglecF^{+}$) and interstitial and recruited macrophages ($CD64^{+}F4/80^{+}SiglecF^{-}$) under homeostasis in WT and $EC^{Sparcl1\text{-}OE}$ mice. n = 4 mice per group, independent biological replicates, gated as shown in Supplementary Figure.11A.

Data in (**A**), (**B**) and (**F**) to (**H**) are presented as means ± SEM, calculated using unpaired two-tailed t test. *P < 0.05, **P < 0.01, ns: not significant, P>0.05. Source data are provided as a Source Data file.

Supplementary Fig.8

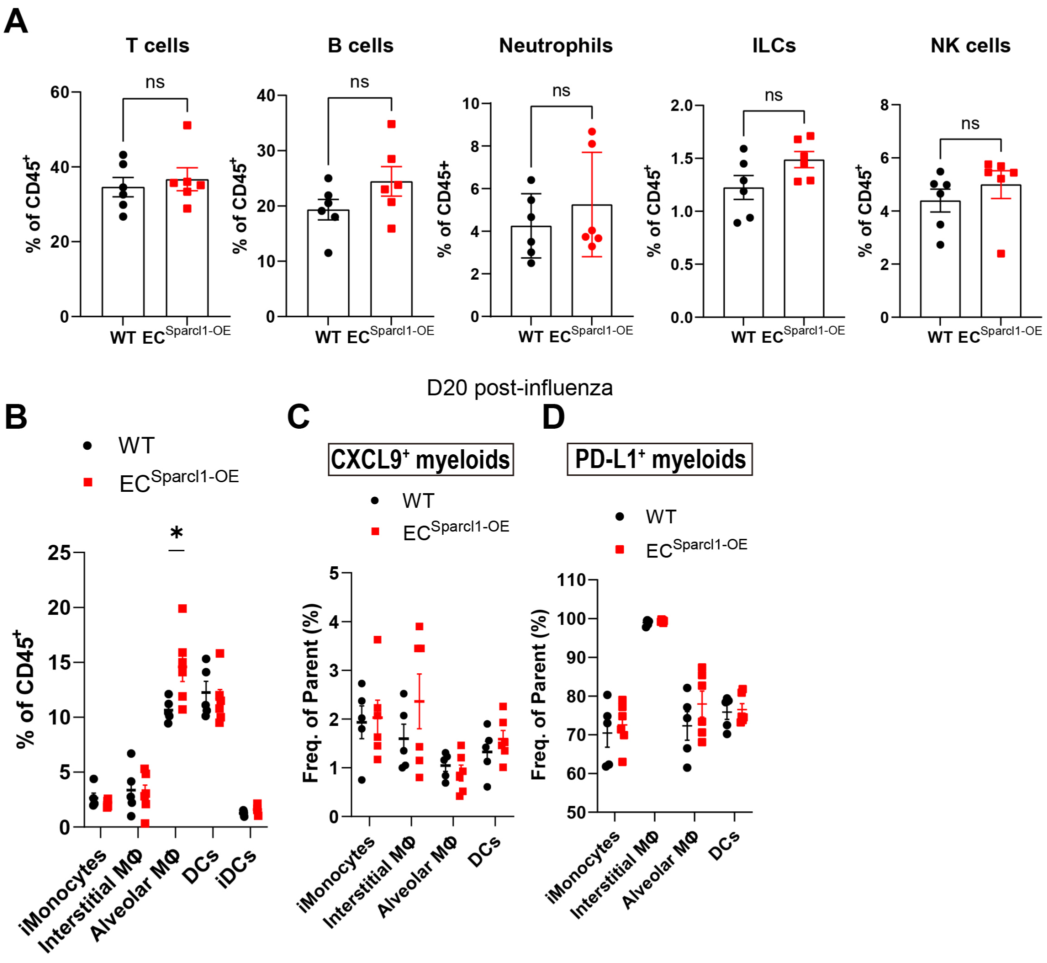

Supplementary Fig.8 The effects of endothelial overexpression of *Sparcl1* on major immune cell populations in the lung on day 20 post-influenza infection.

A. Endothelial overexpression of *Sparcl1* did not affect the frequency of T, B, neutrophils, NK, innate lymphoid cells, gated as shown in Supplementary Figure 6A. n = 5-6 mice per group, independent biological replicates.

B. The effect of endothelial overexpression of *Sparcl1* on the quantity of lung myeloid populations on day 20 after influenza infection, gated as shown in Supplementary Figure 11A, n = 5-6 mice per group, independent biological replicates.

C-D. The effect of endothelial overexpression of *Sparcl1* on myeloids expressing CXCL-9 or PD-L1 on day 12 post infection. Quantification of CXCL-9(C) and PD-L1(D)-expressing myeloids, including inflammatory monocytes (iMonocytes), alveolar macrophages (AMs), interstitial macrophages (IMs) and dendritic cells (DCs), gated as shown in Supplementary Figure 5.

Data in (**A**) to (**D**) are presented as means ± SEM, calculated using unpaired two-tailed t test. *P < 0.05, **P < 0.01, ns: not significant, P>0.05. Source data are provided as a Source Data file.

Supplementary Fig.9

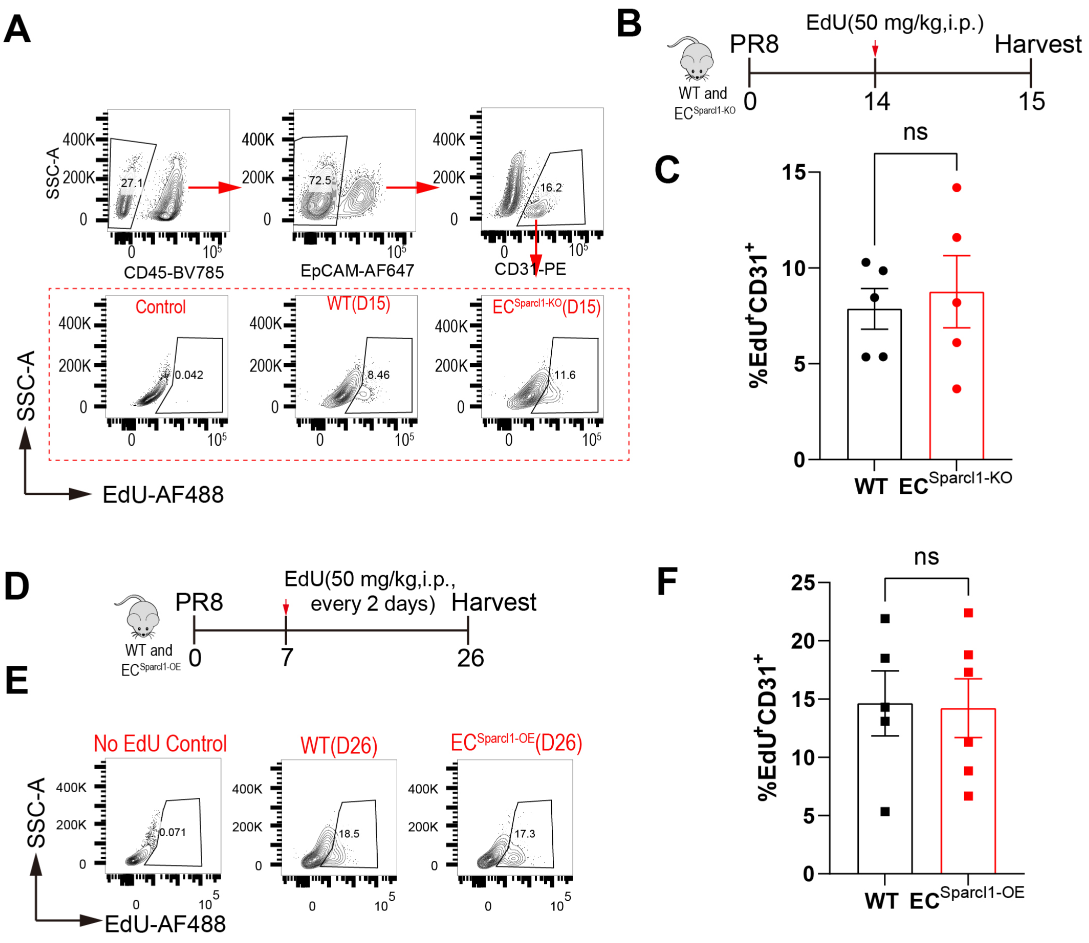

Supplementary Fig.9 SPARCL1 has no obvious effect on endothelial proliferation in influenza-induced lung injury

A. Representative gating scheme for flow cytometry analysis of proliferative endothelial cells ($CD45^{-}EpCAM^{-}CD31^{+}EdU^{+}$) in lungs from WT and *EC^Sparcl1-KO^* mice.

B. Timeline for EdU administration (50 mg/kg, i.p.) and sampling. WT and *EC^Sparcl1-KO^* mice were treated with EdU 24 hour prior to harvest.

C. Endothelial EdU incorporation was measured by flow cytometry in lungs from WT and *EC^Sparcl1-KO^* mice on day 15 after influenza infection, n = 5 mice per group, independent biological replicates, gated as shown in (A).

D. Timeline for EdU administration (50 mg/kg, i.p.) and sampling. WT and *EC^Sparcl1-OE^* mice were treated with EdU on day 7 after influenza infection and every 48 hours per dose later by day 26.

E. Representative gating scheme for flow cytometry analysis of proliferative endothelial cells ($CD45^{-}EpCAM^{-}CD31^{+}EdU^{+}$) in lungs from WT and *EC^Sparcl1-OE^* mice.

F. Endothelial EdU incorporation was measured by flow cytometry in lungs from WT and *EC^Sparcl1-OE^* mice on day 26 after influenza infection, n = 5 mice per group, independent biological replicates, gated as shown in (A) and (E). Source data are provided as a Source Data file.

Data in (**C**) to (**F**) are presented as means ± SEM, calculated using unpaired two-tailed t test. *P < 0.05, **P < 0.01, ns: not significant, P>0.05.

Supplementary Fig.10

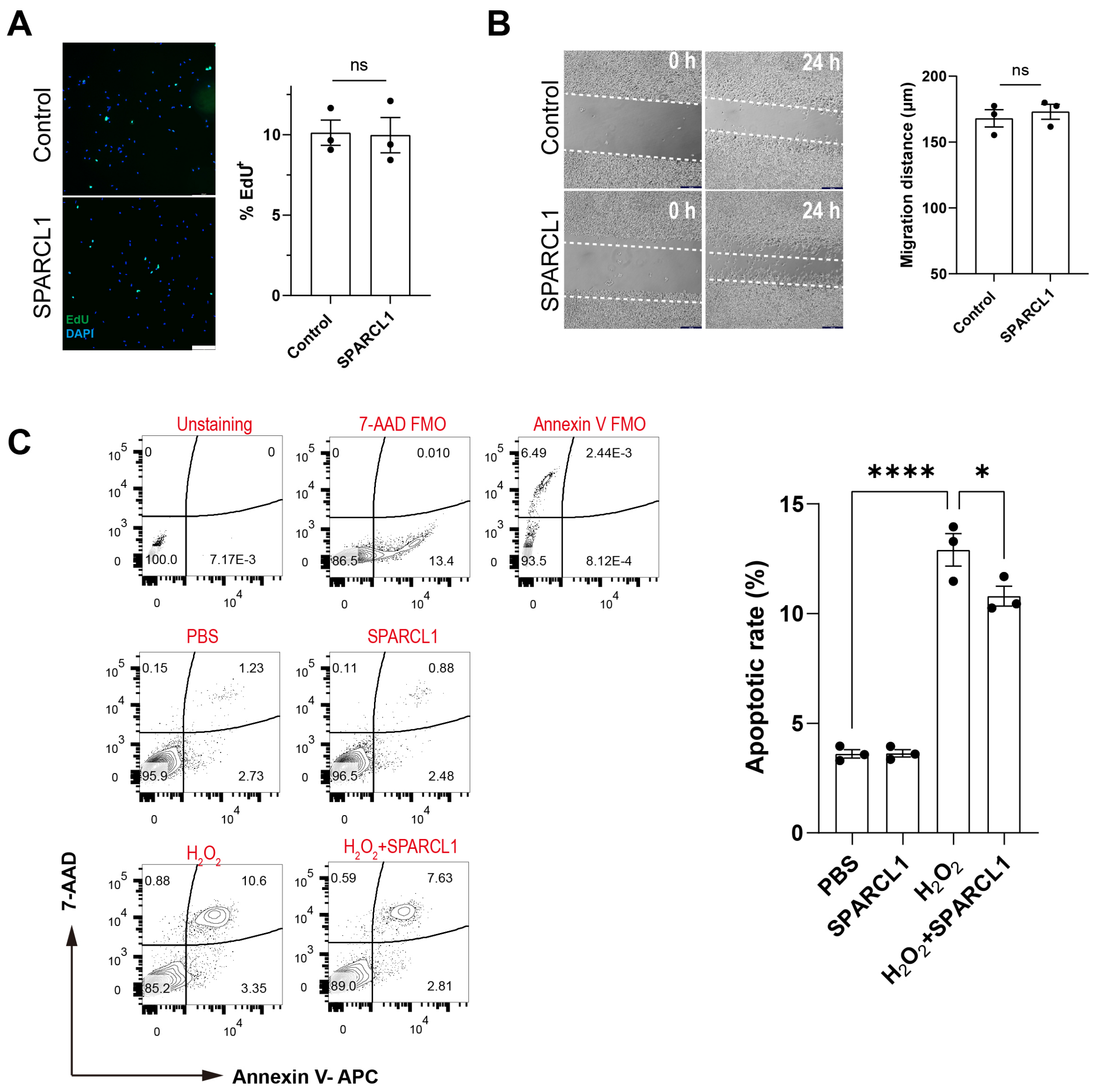

Supplementary Fig.10 The effect of SPARCL1 on macrophage proliferation, migration and apoptosis.

A. BMDMs (Bone Marrow-Derived Macrophages) were treated with recombinant mouse SPARCL1 protein (10 μg/ml, R&D system) for 6 hours and then incubated with EdU for 40 mins. Left: Representative immunofluorescence for the nuclei (blue) and EdU incorporation (green) in WT and SPARCL1 treatment BMDMs, scale bar: 100 μm. Right: quantification of proliferative BMDMs ($EdU^+/DAPI^+$%).

B. BMDMs were treated with or without recombinant mouse SPARCL1 protein (10 μg/ml, R&D system). Cell migration was assessed using a wound scratch assay. Images were obtained at (0 h) and (24 h). Representative photos illustrate scratch closing, quantified in the graph (right), scale bar: 200 μm.

C. BMDMs were pre-treated with or without recombinant mouse SPARCL1 protein (10 μg/ml, R&D system) for 1 hour, following added H_2O_2 (50 μM) or vehicle stimulated for 24 hours. Apoptosis was detected by 7-AAD/annexin V flow cytometry analysis.

Data in (**A**) to (**C**) are presented as means±SEM (n=3 biological replicates), data in (**A**) and (**B**) are calculated using unpaired two-tailed t test. Data in (**C**) are calculated using one-way analysis of variance (ANOVA), followed by Dunnett's multiple comparison test. *$P < 0.05$, and ****$P < 0.0001$. ns: not significant, $P>0.05$. Source data are provided as a Source Data file.

Supplementary Fig.11

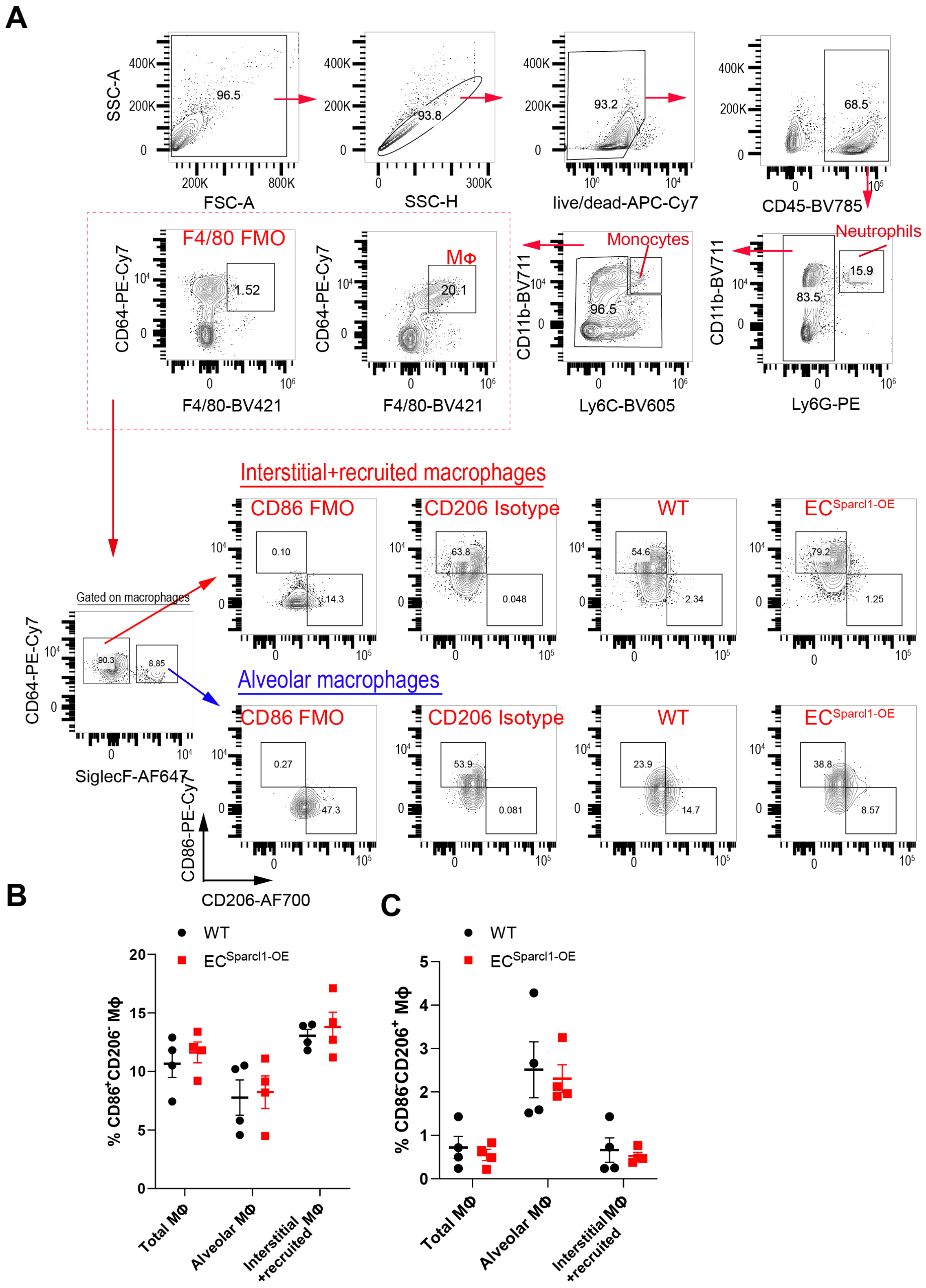

Supplementary Fig.11 Endothelial overexpression of Sparcl1 did not significantly alter the M1/M2 macrophage transition at homeostasis.

A. Representative gating scheme for identification of general pulmonary macrophages ($CD45^{+}Ly6G^{-}CD64^{+}F4/80^{+}$) and M1-like ($CD86^{+}CD206^{-}$) and M2-like ($CD86^{-}CD206^{+}$) macrophages in total lung macrophages ($CD45^{+}Ly6G^{-}CD64^{+}F4/80^{+}$), alveolar macrophages ($CD45^{+}Ly6G^{-}CD64^{+}F4/80^{+}SiglecF^{+}$) and interstitial and recruited macrophages ($CD45^{+}Ly6G^{-}CD64^{+}F4/80^{+}SiglecF^{-}$) at day 20 after influenza infection in WT and $EC^{Sparcl1\text{-}OE}$ mice.

B-C. Quantification of the proportion of (**B**)M1-like ($CD86^{+}CD206^{-}$) and (**C**)M2-like ($CD86^{-}CD206^{+}$) macrophages in total lung macrophages ($CD45^{+}Ly6G^{-}CD64^{+}F4/80^{+}$), alveolar macrophages ($CD45^{+}Ly6G^{-}CD64^{+}F4/80^{+}SiglecF^{+}$) and interstitial and recruited macrophages ($CD45^{+}Ly6G^{-}CD64^{+}F4/80^{+}SiglecF^{-}$) at day 0 after influenza infection (uninjured) in WT and $EC^{Sparcl1\text{-}OE}$ mice, n = 4 mice per group, independent biological replicates, gated as shown in (A). Data in (**B**) to (**C**) are presented as means ± SEM, calculated using unpaired two-tailed t test. *P < 0.05. Source data are provided as a Source Data file.

Supplementary Fig.12

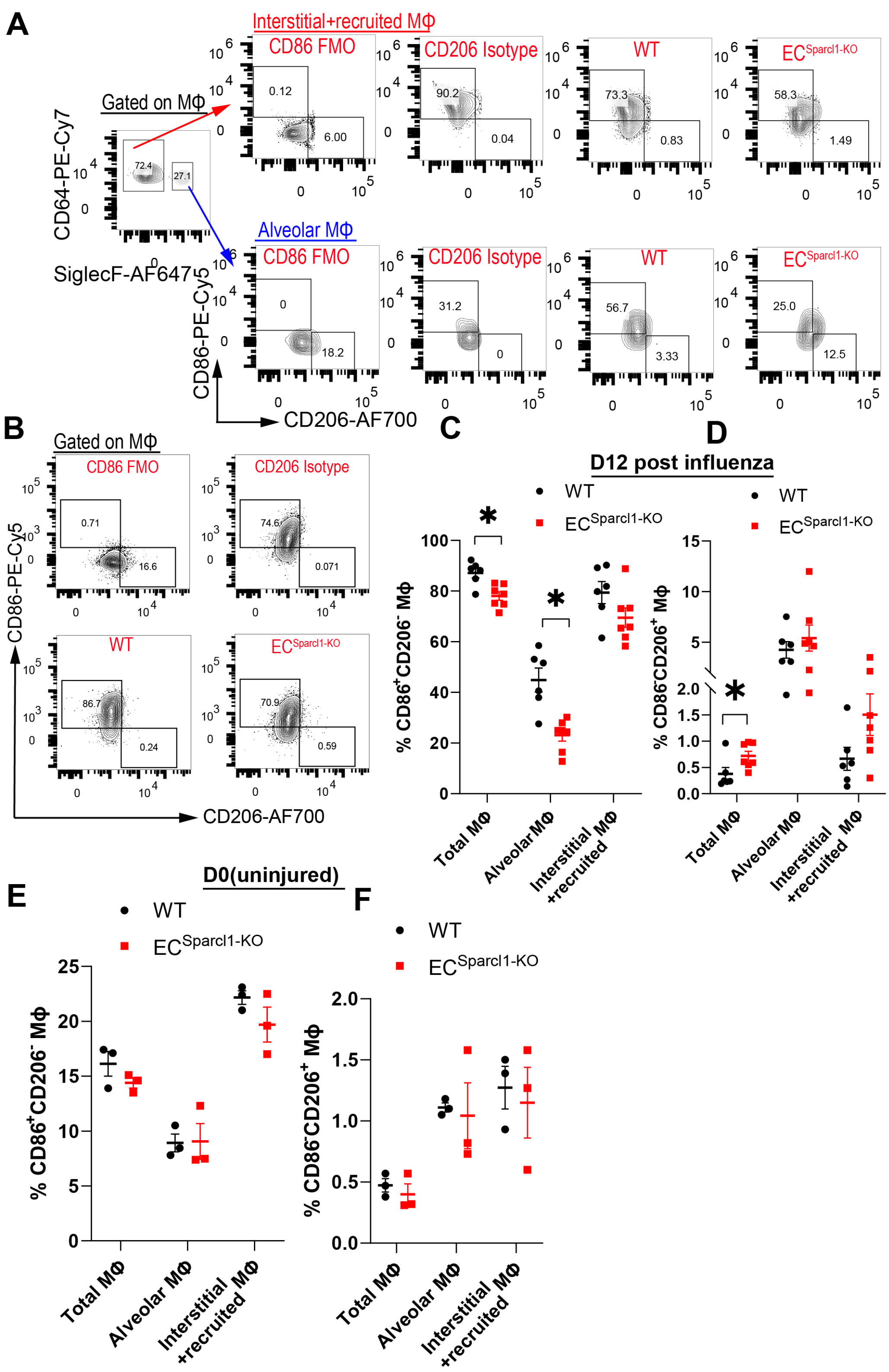

Supplementary Fig.12 Endothelial loss of Sparcl1 reduced M1_like but enhanced M2_like macrophages during pneumonia

A. Representative gating scheme for identification of M1-like ($CD86^{+}CD206^{-}$) and M2-like ($CD86^{-}CD206^{+}$) macrophages in alveolar macrophages ($CD45^{+}Ly6G^{-}CD64^{+}F4/80^{+}SiglecF^{+}$) and interstitial and recruited macrophages ($CD45^{+}Ly6G^{-}CD64^{+}F4/80^{+}SiglecF^{-}$) at day 12 after influenza infection in WT and $EC^{Sparcl1\text{-}KO}$ mice.

B. Representative gating scheme for identification of M1-like ($CD86^{+}CD206^{-}$) and M2-like ($CD86^{-}CD206^{+}$) macrophages in total lung macrophages ($CD45^{+}Ly6G^{-}CD64^{+}F4/80^{+}$) at day 12 after influenza infection in WT and $EC^{Sparcl1\text{-}KO}$ mice.

C-D. Quantification of the proportion of (**C**)M1-like ($CD86^{+}CD206^{-}$) and (**D**)M2-like ($CD86^{-}CD206^{+}$) macrophages in total lung macrophages ($CD45^{+}Ly6G^{-}CD64^{+}F4/80^{+}$), alveolar macrophages ($CD45^{+}Ly6G^{-}CD64^{+}F4/80^{+}SiglecF^{+}$) and interstitial and recruited macrophages ($CD45^{+}Ly6G^{-}CD64^{+}F4/80^{+}SiglecF^{-}$) at day 12 after influenza infection in WT and $EC^{Sparcl1\text{-}KO}$ mice, n = 6-7 mice per group, independent biological replicates, gated as shown in (A-B).

E-F. Quantification of the proportion of (**E**)M1-like ($CD86^{+}CD206^{-}$) and (**F**)M2-like ($CD86^{-}CD206^{+}$) macrophages in total lung macrophages ($CD45^{+}Ly6G^{-}CD64^{+}F4/80^{+}$), alveolar macrophages ($CD45^{+}Ly6G^{-}CD64^{+}F4/80^{+}SiglecF^{+}$) and interstitial and recruited macrophages ($CD45^{+}Ly6G^{-}CD64^{+}F4/80^{+}SiglecF^{-}$) at day 0 after influenza infection (uninjured) in WT and $EC^{Sparcl1\text{-}KO}$ mice, n = 3 mice per group, independent biological replicates , gated as shown in (A-B).

Data in (**C**) to (**F**) are presented as means ± SEM, calculated using unpaired two-tailed t test. $^{*}P < 0.05$. Source data are provided as a Source Data file.

Supplementary Fig.13

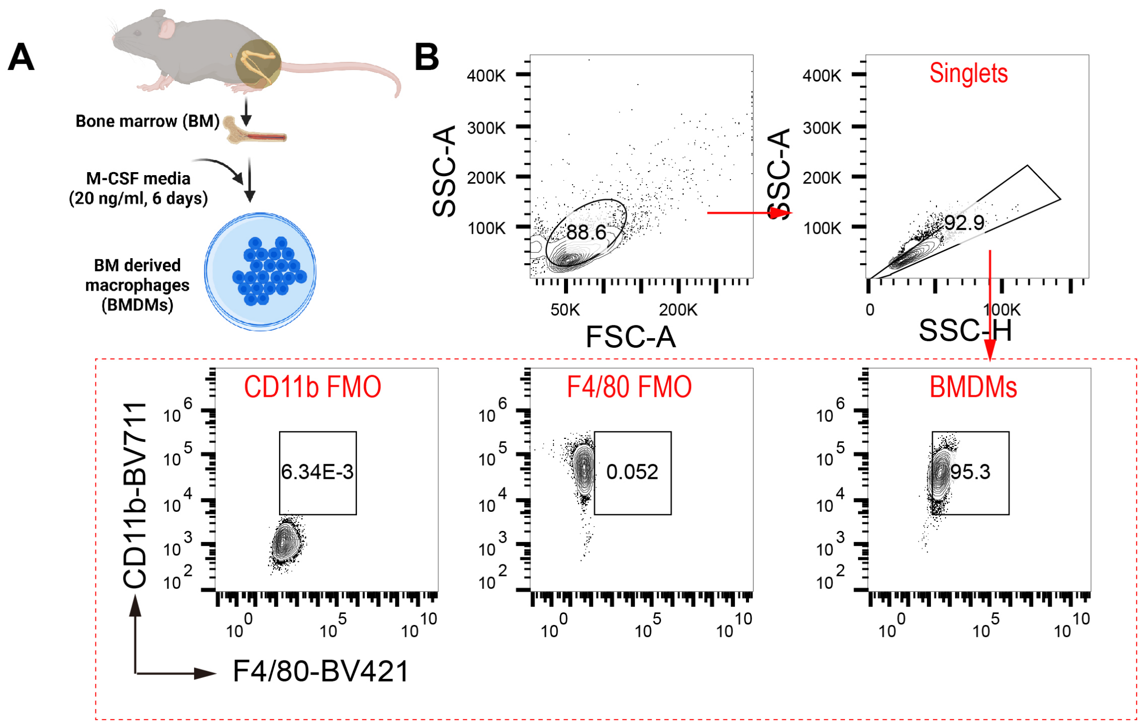

Supplementary Fig.13 Isolation and differentiation of bone marrow (BM) cells to BM-derived macrophages (BMDMs).

A. Schematic for differentiation of bone marrow cells into bone marrow-derived macrophages (BMDMs).

B. FACS analysis of BMDM cell suspension. Over 95% of the cells were positive for CD11b and F4/80, markers for murine macrophages[5]. Source data are provided as a Source Data file. Schematics and icons created with BioRender.com.

Supplementary Fig.14

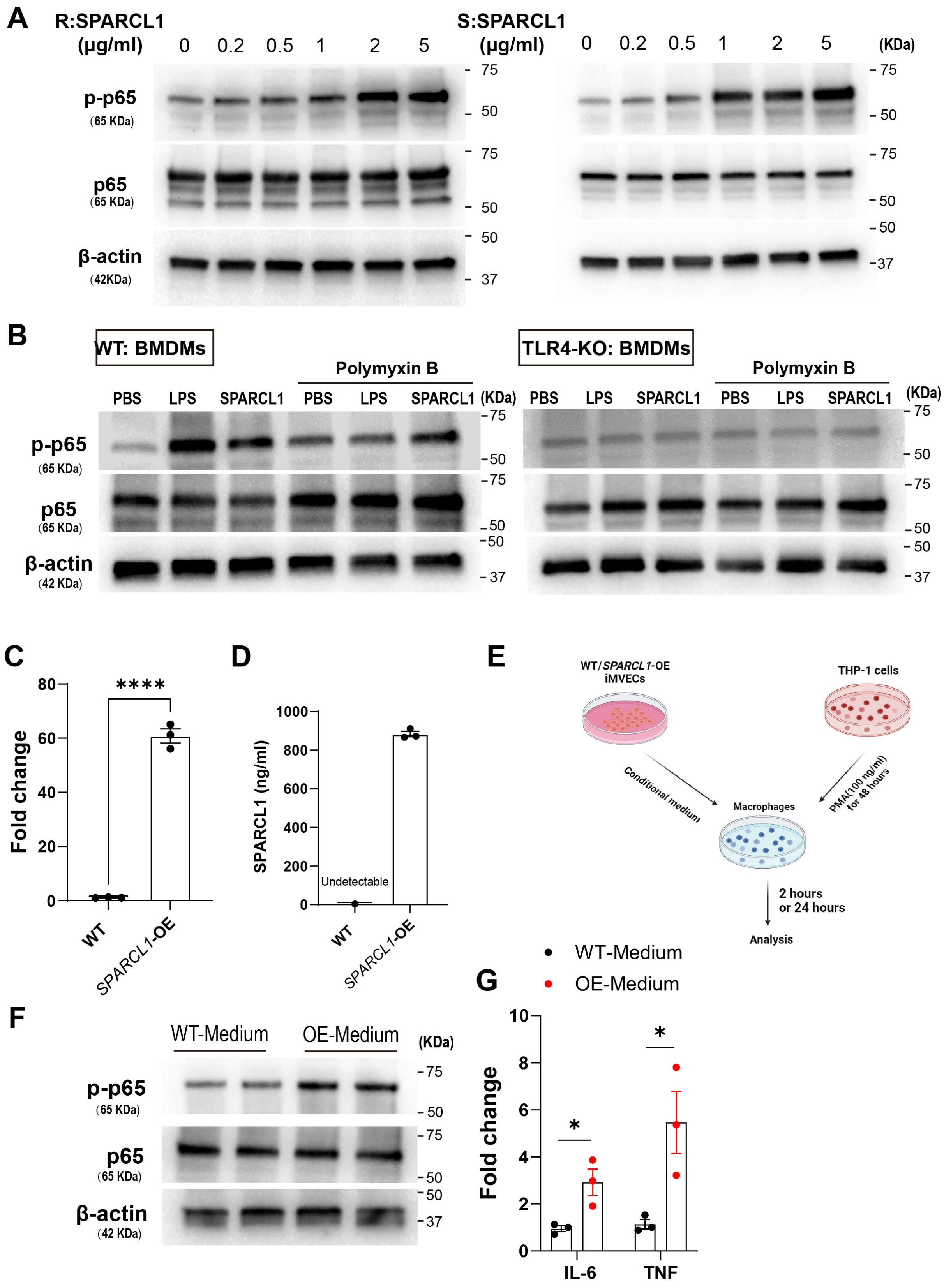

Supplementary Fig.14 SPARCL1 induces NF-κB P65 phosphorylation
A. BMDMs were exposed to varying doses (0, 0.2, 0.5, 1, 2, and 5 μg/ml) of recombinant SPARCL1 protein for 90 mins. Subsequently, immunoblot analysis was conducted to evaluate the phosphorylation of NF-κB p65. Left: Recombinant protein purchased from R&D Systems; Right: Recombinant protein purchased from Sino Biological.
B. BM cells were isolated from WT (C57BL/6) and *Tlr4*$^{-/-}$ (TLR4-KO) mice and differentiated into BMDMs. Subsequently, they were pre-treated with or without Polymyxin (100 μg/ml) for 1 hour, followed by stimulation with LPS (50 ng/ml), recombinant SPARCL1 protein (10 μg/ml, Sino Biological), or vehicle PBS for 90 mins. The cells were then subjected to immunoblot analysis to evaluate NF-κB p65 phosphorylation.
C. qPCR was performed to analyze the expression levels of *SPARCL1* in SPARCL1-OE iMVECs with SPARCL1 overexpression.
D. ELISA measurement of SPARCL1 concentration in supernatants collected from WT and SPARCL1-OE iMVECs.
E-G. **(E),** Experimental design diagram: THP-1 cells, induced with PMA (100 ng/ml) for 48 hours to differentiate into macrophages. Subsequently, treated with supernatants from WT or SPARCL1-OE iMVECs for 2 hours (collect cells for immunoblot analysis) or 24 hours (collect cells for qPCR). (**F**), Western blotting analysis for the phosphorylation of NF-κB p65. (**G**), qPCR analysis for *TNF* and *IL-6*.
Data in (**C**), (**D**) and (**G**) are presented as means±SEM (n=3 biological replicates), calculated using unpaired two-tailed t test. *P < 0.05, ****P <0.0001. Source data are provided as a Source Data file. Schematics and icons created with BioRender.com.

Supplementary Fig.15

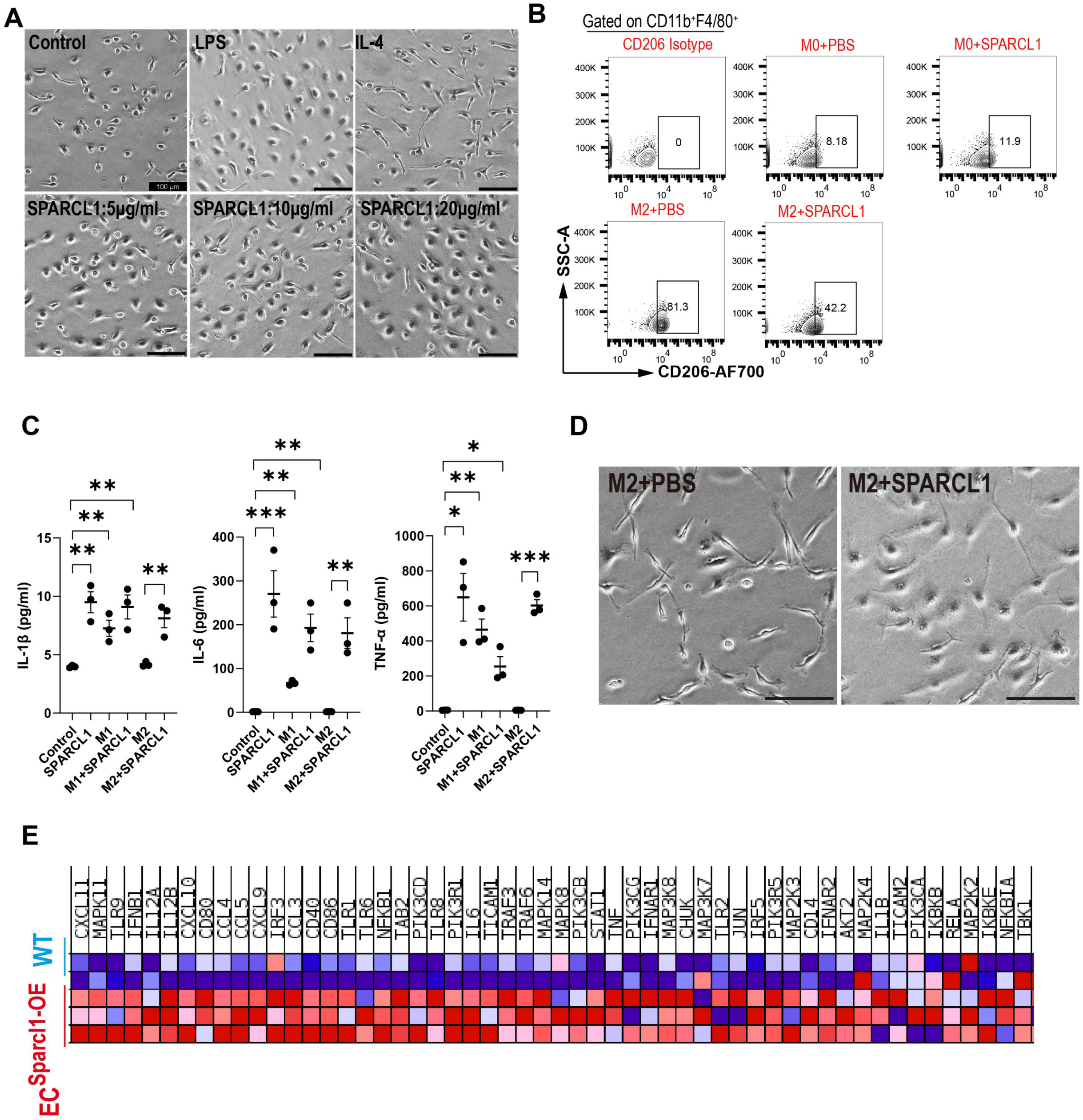

Supplementary Fig.15 SPARCL1 induces an M1-like macrophage phenotype in BMDMs
A. BMDMs were treated with lipopolysaccharide (LPS, 50 ng/ml), IL-4 (20 ng/ml), SPARCL1 (5, 10 and 20 μg/ml), or vehicle control for 24 hours. Cell morphology was observed and imaged by light microscopy. Scale bar: 100 μm.
B. Representative gating scheme for M2-like (F4/80^{+}CD206^{+}) macrophages after SPARCL1 treatment.**C**. ELISA quantification of Th1 cytokines IL-1β, IL-6 and TNF levels in supernatant collected from M1 and M2 polarized BMDMs after treatment with SPARCL1(10 μg/ml) for 24 hours, n = 3 per group.
D. Cell morphology was observed 24 h after treatment of M2-polarized BMDM with SPARCL1 (10 μg/ml). Scale bar: 100 μm.
E. Heatmaps for genes in the TLR4 signaling pathway that positively correlated to endothelial Sparcl1 expression are shown.
Data in (**C**) are presented as means ± SEM (n = 3 biological replicates), calculated using unpaired two-tailed t test. *P < 0.05. **P < 0.01, ***P <0.001. Source data are provided as a Source Data file.

Supplementary Fig.16

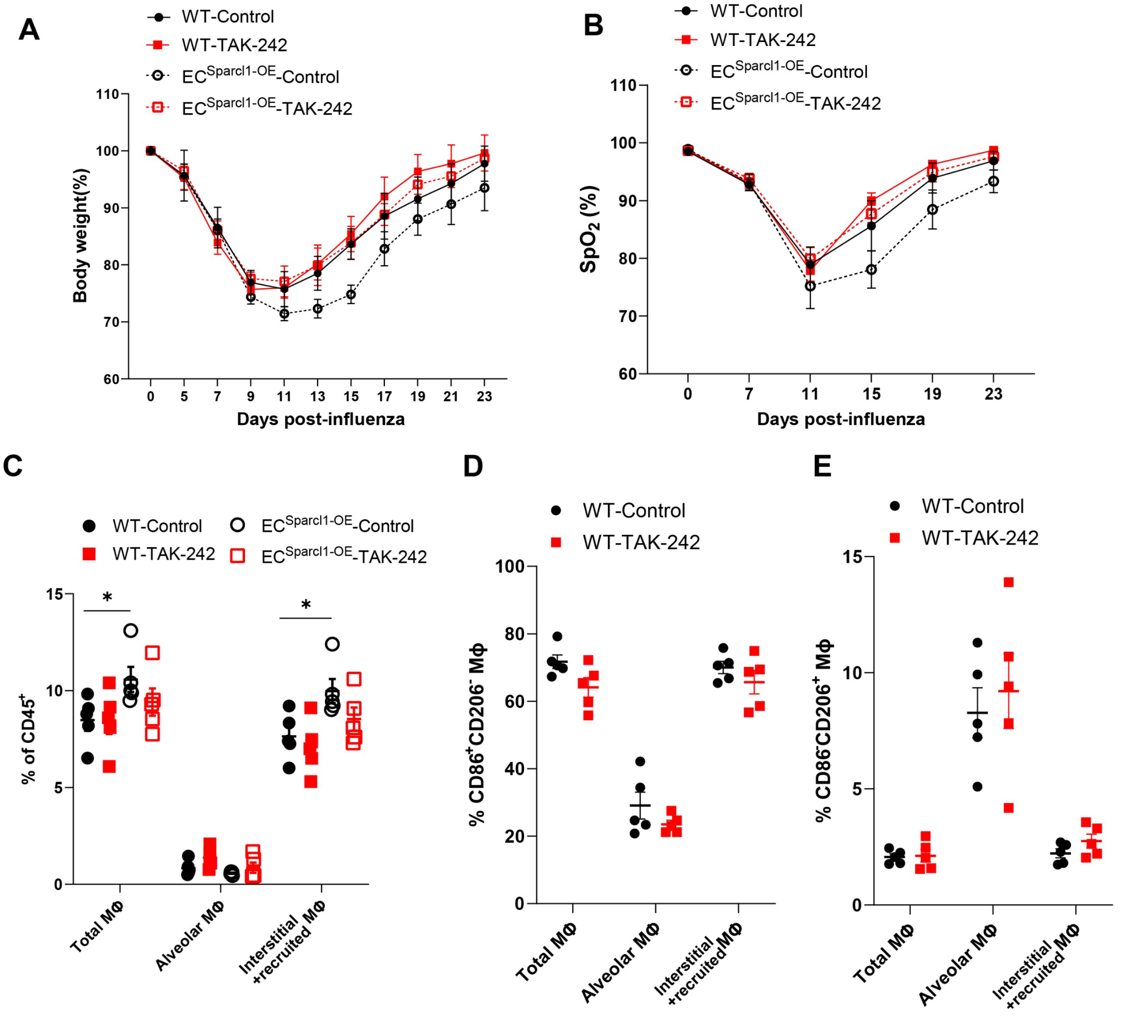

Supplementary Fig.16 TAK-242 treatment did not significantly improve influenza-induced pneumonia in WT mice

A-B. Time course of changes in (**A**) body weight and (**B**) capillary oxygen saturation in WT and EC$^{Sparcl1-OE}$ mice treated with or without TAK-242 after influenza infection, n = 5 mice per group.
C. Quantification of total lung macrophages (CD64$^+$F4/80$^+$), alveolar macrophages (CD64$^+$F4/80$^+$SiglecF$^+$) and interstitial and recruited macrophages (CD64$^+$F4/80$^+$SiglecF$^-$) in WT and EC$^{Sparcl1-OE}$ mice treated with or without TAK-242 on day 20 after influenza infection, n = 5 mice per group, independent biological replicates, gated as shown in Supplementary Figure 11A.
D-E. Quantification of the proportion of (**C**)M1-like (CD86$^+$CD206$^-$) and (**D**)M2-like (CD86$^-$CD206$^+$) macrophages in total lung macrophages (CD45$^+$Ly6G$^-$CD64$^+$F4/80$^+$), alveolar macrophages (CD45$^+$Ly6G$^-$CD64$^+$F4/80$^+$SiglecF$^+$) and interstitial and recruited macrophages (CD45$^+$Ly6G$^-$CD64$^+$F4/80$^+$SiglecF$^-$) at day 20 after influenza infection in WT mice treated with or without TAK-242, n = 5 mice per group, independent biological replicates, gated as shown in Supplementary Figure 11A.

Data in (**A**) to (**E**) are presented as means ± SEM, data in (**C**) are calculated using one-way analysis of variance (ANOVA), followed by Dunnett's multiple comparison test. $*P < 0.05$. Source data are provided as a Source Data file.

Tables

Supplementary Table 1.

**mSPARCL1 gRNA sequences used for ES cells (5'-3')**	
gRNA1	F: caccTCTGCCTACTCCTATGCATG
	R: aaacCATGCATAGGAGTAGGCAGA
gRNA2	F:accgTATATATACCACTTGCACTTgt
	R:taaaacAAGTGCAAGTGGTATATATA

Sequences of primers used for qPCR

Species	gene name	sequences(5'-3')
mouse	*Sparcl1*	F:TGAAGGCTGTGCTTCTCCTCC
		R:GGTTGGAGTGGTCAGAGAGAAA
	Mrc1	F:CTCTGTTCAGCTATTGGACGC
		R:CGGAATTTCTGGGATTCAGCTTC
	Chil3	F:CAGGTCTGGCAATTCTTCTGAA
		R:GTCTTGCTCATGTGTGTAAGTGA
	RL19	F:ATGTATCACAGCCTGTACCTG
		R:TCCTTGGTCTCTTCCTCCTTG
human	*SPARCL1*	F:CCAACTGAAGGTACATTGGACAT
		R:CTGTGAAGGAACTAACACCAGG
	TNF	F:CCTCTCTCTAATCAGCCCTCTG
		R:GAGGACCTGGGAGTAGATGAG
	IL6	F:ACTCACCTCTTCAGAACGAATTG
		R:CCATCTTTGGAAGGTTCAGGTTG
	GAPDH	F:TGGTATCGTGGAAGGACTCATGAC
		R:ATGCCAGTGAGCTTCCCGTTCAGC
Influeza M-gene		F:TCAGGCCCCCTCAAAGCCGAG
		R:CGTCTACGCTGCAGTCCTC

Sequences of primers used for genotyping		
$Sparcl1^{flox}$	WT allele	F: ACTCCTATGCATGAGGCT<br>R: CTGAAGGACAATGCTCCCAA
	Mutant allele	F: GAAGTAAGAACTGAAATAACTCTGC<br>R: TCCACTCTCCAGGTGACTA
R26-LSL-Sparcl1	WT allele	F: GTGGGAAGTCTTGTCCCTCC<br>R: CCTCTTCCCTCGTGATCTGC
	Mutant allele	F: GGGGCCACTGCTTTGGAATTA<br>R: AACTAGAAGGCACAGTCGAGGC

PCR program for genotyping ($Sparcl1^{flox}$ and R26-LSL-Sparcl1)		
STEP	TEMP	TIME
Initial Denaturation	95°C	4 min
35X cycles	95°C	30 sec
	56°C	30 sec
	72°C	20 sec
Final extension	72°C	10 min
Hold	4°C	∞

Supplementary Table 1. Primer and guide RNA sequences, genotyping information.

Supplementary Table 2.

**Supplementary Table: Clinical characteristics**	COVID-19 non-survivors (n=28)	COVID-19 survivors (n=131)
Age in years	68.4 ± 2.2	57.4 ± 1.2
Female sex	11 (39%)	61 (47%)
Race		
Black	15 (54%)	92 (69%)
White	9 (32%)	34 (26%)
Asian	3 (11%)	4 (3%)
Other	1 (4%)	3 (2%)
Hispanic ethnicity	1 (4%)	11 (8%)
APACHE III score	82.4 ± 18.4	56.6 ± 27.2
Comorbidity		
Hypertension	20 (71%)	89 (68%)
Diabetes	12 (43%)	46 (35%)
Cardiovascular disease	9 (32%)	15 (11%)
Heart failure	4 (14%)	22 (17%)
Renal insufficiency	8 (29%)	24 (18%)
WHO ordinal scale at the time of blood draw		
4: hospitalized, not requiring O2	1 (3%)	31 (24%)
5: hospitalized, requiring O2 ≤ 6 lpm	3 (11%)	39 (29%)
6: hospitalized, requiring high flow O2 or non-invasive ventilation	8 (29%)	31 (24%)
7: hospitalized, requiring invasive ventilation	16 (57%)	30 (23%)

Supplementary Table 2. Clinical characteristics of the COVID-19 cohort. Characteristics were abstracted from the electronic health record and asking participants or their proxies to self-identify their race and ancestry. Survival was adjudicated at 90 days. *APACHE:* acute physiology and chronic health evaluation. *WHO:* World Health Organization.

References

1. Kalucka J, *et al.* Single-Cell Transcriptome Atlas of Murine Endothelial Cells. *Cell* **180**, 764-779 e720 (2020).

2. Gillich A, *et al.* Capillary cell-type specialization in the alveolus. *Nature* **586**, 785-789 (2020).

3. Zhou Y, *et al.* Metascape provides a biologist-oriented resource for the analysis of systems-level datasets. *Nat Commun* **10**, 1523 (2019).

4. Schupp JC, *et al.* Integrated Single-Cell Atlas of Endothelial Cells of the Human Lung. *Circulation* **144**, 286-302 (2021).

5. Toda G, Yamauchi T, Kadowaki T, Ueki K. Preparation and culture of bone marrow-derived macrophages from mice for functional analysis. *STAR Protoc* **2**, 100246 (2021).
