# Supplementary material for: Vascular endothelial-derived SPARCL1 exacerbates viral pneumonia through pro-inflammatory macrophage activation
Source: Nat Commun. 2024 May 18;15:4235. doi: 10.1038/s41467-024-48589-3 (PMC11102455; doi:10.1038/s41467-024-48589-3)
Supplement: Supplementary file 3 — Reporting Summary [file 41467_2024_48589_MOESM3_ESM.pdf]

nature portfolio

Corresponding author(s): Andrew Vaughan, Gan Zhao

Last updated by author(s): Mar 30, 2024

Reporting Summary

Nature Portfolio wishes to improve the reproducibility of the work that we publish. This form provides structure for consistency and transparency in reporting. For further information on Nature Portfolio policies, see our Editorial Policies and the Editorial Policy Checklist.

Statistics

For all statistical analyses, confirm that the following items are present in the figure legend, table legend, main text, or Methods section.

n/a	Confirmed	
☐	☒	The exact sample size (*n*) for each experimental group/condition, given as a discrete number and unit of measurement
☐	☒	A statement on whether measurements were taken from distinct samples or whether the same sample was measured repeatedly
☐	☒	The statistical test(s) used AND whether they are one- or two-sided<br>*Only common tests should be described solely by name; describe more complex techniques in the Methods section.*
☐	☒	A description of all covariates tested
☐	☒	A description of any assumptions or corrections, such as tests of normality and adjustment for multiple comparisons
☐	☒	A full description of the statistical parameters including central tendency (e.g. means) or other basic estimates (e.g. regression coefficient) AND variation (e.g. standard deviation) or associated estimates of uncertainty (e.g. confidence intervals)
☐	☒	For null hypothesis testing, the test statistic (e.g. *F*, *t*, *r*) with confidence intervals, effect sizes, degrees of freedom and *P* value noted<br>*Give P values as exact values whenever suitable.*
☒	☐	For Bayesian analysis, information on the choice of priors and Markov chain Monte Carlo settings
☒	☐	For hierarchical and complex designs, identification of the appropriate level for tests and full reporting of outcomes
☒	☐	Estimates of effect sizes (e.g. Cohen's *d*, Pearson's *r*), indicating how they were calculated

Our web collection on statistics for biologists contains articles on many of the points above.

Software and code

Policy information about availability of computer code

Data collection	FACS Diva(V9.0 BD), LAS X software (Leica), MouseOx® Plus Software (V1.0 STARR Life Sciences Corp.) , Gen5 Software(Bio Tek), ChemiDoc MP Imaging System(Bio Rad)
Data analysis	Fluorescent microscopy imaging data were analyzed using LAS X software (Leica). All statistical calculations were performed using GraphPad Prism 9.0 (GraphPad Software Inc.) Flow cytometry results were analyzed by FACS and FlowJo v10. Capillary blood oxygen was measured by MouseOx® Plus Small Animal Vital Signs Monitor and recorded by MouseOx® Plus Software (STARR Life Sciences Corp.).Single-cell sequencing was performed on a 10X Chromium instrument (10X Genomics). Cellranger mkfastq was used to generate demultiplexed FASTQ files from the raw sequencing data. Next, Cellranger count was used to align sequencing reads to the mouse reference genome (GRCm38) and generate single cell gene barcode matrices. Post processing and secondary analysis was performed using the Seurat package (v.4.0). For Bulk-RNA seq analysis, raw data (fastq.gz) were processed through a general pipeline. Reads were aligned to the mm10 mouse genome using Kallisto and imported into R Studio for analysis via the TxImport package. Volcano plots were created using the OmicStudio tools at https://www.omicstudio.cn/tool. All detectable genes derived from RNA-seq were used for gene set enrichment analysis (GSEA) using the Molecular Signatures Database (MSigDB) C2: curated gene sets according to the standard GSEA user guide (http://www.broadinstitute.org/gsea/doc/GSEAUserGuideFrame.html).

For manuscripts utilizing custom algorithms or software that are central to the research but not yet described in published literature, software must be made available to editors and reviewers. We strongly encourage code deposition in a community repository (e.g. GitHub). See the Nature Portfolio guidelines for submitting code & software for further information.

Data

Policy information about availability of data

All manuscripts must include a data availability statement. This statement should provide the following information, where applicable:

- Accession codes, unique identifiers, or web links for publicly available datasets
- A description of any restrictions on data availability
- For clinical datasets or third party data, please ensure that the statement adheres to our policy

All data are included in the Supplementary Information or available from the authors, as are unique reagents used in this Article. The raw numbers for charts and graphs are available in the Source Data file whenever possible. The sequencing data have been deposited in GEO under the accession code GSE201631 and GSE225439.

Research involving human participants, their data, or biological material

Policy information about studies with human participants or human data. See also policy information about sex, gender (identity/presentation), and sexual orientation and race, ethnicity and racism.

Reporting on sex and gender	Details see table S2
Reporting on race, ethnicity, or other socially relevant groupings	Details see table S2
Population characteristics	Details see table S2
Recruitment	COVID-19 tissue samples were from patients who previously tested positive for COVID-19 by PCR but tested negative via PCR multiple times prior to tissue acquisition. All COVID-19 samples were obtained from ventilated ARDS patients at least 30 days post-hospitalization who underwent lung transplant, at which time tissue samples were acquired. Human plasma samples were from a prospective cohort study of participants with high risk for sepsis (Table S2) as approved by the University of Pennsylvania Institutional Review Board Protocol #808542. All human participants or their proxies provided written informed consent to participate. Participants were characterized by the World Health Organization ordinal scale for respiratory failure 56 at the time of blood sampling and considered severe respiratory failure if they required high flow oxygen (>6 lpm), non-invasive ventilation, or invasive ventilation (WHO ordinal scale ≥ 6) and moderate respiratory injury if they required no oxygen or oxygen at flow rates at or below 6 lpm (WHO ordinal scale ≤ 5). Mortality was assessed at 90 days using the EHR, which included a surveillance program post-discharge for patients discharged after COVID-19.
Ethics oversight	University of Pennsylvania Institutional Review Board

Note that full information on the approval of the study protocol must also be provided in the manuscript.

Field-specific reporting

Please select the one below that is the best fit for your research. If you are not sure, read the appropriate sections before making your selection.

☒ Life sciences ☐ Behavioural & social sciences ☐ Ecological, evolutionary & environmental sciences

For a reference copy of the document with all sections, see nature.com/documents/nr-reporting-summary-flat.pdf

Life sciences study design

All studies must disclose on these points even when the disclosure is negative.

Sample size	All in vitro experiments were repeated at least 3 times unless otherwise stated. All in vivo experiments were performed using a minimum of 5 mice per group. The number of mice and statistical methods used for individual in vivo experiments is specified in the figure legends.
Data exclusions	No data was excluded from the analysis.
Replication	Experiments were repeated at least three independent experiments with similar results. All experiments were reproduced to reliably support conclusions stated in the manuscript.
Randomization	Cages of mice were randomly selected and then divided into experimental groups for further in vivo dosing treatment. Groups in all the in vitro and in vivo experiments were selected randomly.
Blinding	Investigators were blinded to group allocation during experiments.

Reporting for specific materials, systems and methods

We require information from authors about some types of materials, experimental systems and methods used in many studies. Here, indicate whether each material, system or method listed is relevant to your study. If you are not sure if a list item applies to your research, read the appropriate section before selecting a response.

Materials & experimental systems

n/a	Involved in the study
☐	☒ Antibodies
☐	☒ Eukaryotic cell lines
☒	☐ Palaeontology and archaeology
☐	☒ Animals and other organisms
☐	☒ Clinical data
☒	☐ Dual use research of concern
☒	☐ Plants

Methods

n/a	Involved in the study
☒	☐ ChIP-seq
☐	☒ Flow cytometry
☒	☐ MRI-based neuroimaging

Antibodies

Antibodies used

Flow antibodies:
allophycocyanin (APC)/Cyanine7 or Brilliant Violet 421™ anti-human CD45 antibody (1:200, Biolegend, HI30), APC anti-human CD31 antibody (1:200, Biolegend, WM59) and PE anti-human CD326 (EpCAM) antibody (1:200, Biolegend, 9C4) for human lungs; Brilliant Violet 785™ anti-mouse CD45 antibody (1:200; Biolegend, 30-F11), Brilliant Violet 711™ anti-mouse/human CD11b antibody (1:200; Biolegend, M1/70), Brilliant Violet 421™ anti-mouse F4/80 antibody(1:100; Biolegend, BM8), Alexa Fluor® 647 anti-mouse Siglec-F antibody (1:100; BD Bioscience, E50-2440), PE anti-mouse Ly-6G antibody(1:200; Biolegend, 1A8), PE/Cyanine7 anti-mouse CD64 (FcγRI) antibody(1:200, Biolegend, X54-5/7.1), Alexa Fluor 488 or PE–conjugated rat anti-mouse CD31 [platelet endothelial cell adhesion molecule 1 (PECAM1)] antibody (1:200; BioLegend, MEC13.3), Alexa Fluor 647 or FITC–conjugated rat anti-mouse CD326 (Ep-CAM) antibody(1:200; Biolegend, G8.8), PE/Cyanine5 anti-mouse CD86 antibody(1:200; Biolegend, GL-1), PE/Cyanine5 anti-mouse CD3ε antibody (1:200; Biolegend, 145-2C11), Alexa Fluor 700 anti-mouse/human CD45R/B220 antibody(1:50; Biolegend, RA3-6B2), BUV395 anti-mouse CD11b(1:200; BD Biosciences, M1/70), Brilliant Violet 421™ anti-mouse NK-1.1 antibody(1:100; Biolegend, PK136), PE-Cyanine7 CD127 Monoclonal antibody (1:100; Invitrogen, A7R34), Alexa Fluor® 700 anti-mouse CD206 (MMR) antibody (1:200; Biolegend, C068C2) ,RELM alpha monoclonal antibody (DS8RELM), Alexa Fluor™ 700(1:200; Invitrogen, #56-5441-82), PE anti-mouse CXCL9 (MIG) antibody (1:100; Biolegend, #515603), BD Horizon™ BUV395 Rat Anti-Mouse CD45(1:200; BD Bioscience, 30-F11), BD Horizon™ BUV563 Rat Anti-Mouse Ly-6G(1:200; BD Bioscience, 1A8), Brilliant Violet 605™ anti-mouse Ly-6C antibody (1:100; Biolegend, HK1.4), FITC anti-mouse CD11c antibody (1:100; Biolegend, N418), PE/Cyanine5 anti-mouse I-A/I-E antibody (1:1000; Biolegend, M5/114.15.2), Brilliant Violet 785™ anti-mouse CD274 (B7-H1, PD-L1) antibody (1:200; Biolegend, 10F.9G2).
Immunostaining antibodies:
CD31 1:200, Biolegend, MEC13.3; mSPARCL1 1:500, R&D systems, #AF2836-SP; hSPARCL1 1:500, R&D systems, #AF2728-SP; ERG 1:2000, Abcam, #ab92513; F4/80 1:200, Cell Signaling Technology, #30325; RELMα 1:200; Invitrogen, #56-5441-82; α-SMA 1:1000, Abcam, # ab32575; and the fluorophore-conjugated secondary antibodies (typically Alexa Fluor conjugates, Life Sciences) at a 1:1000 dilution.
Western blotting antibodies:
phospho-NF-κB p65 1:1000, Cell Signaling Technology, #3033; NF-κB p65 1:1000, Cell Signaling Technology, #8242; mSPARCL1 1:500, R&D systems, #AF2836-SP; β-actin 1:2000, Cell Signaling Technology, #4970

Validation

All antibodies were used in the study according to the profile of manufactures. all other details are provided in the manuscript.

Eukaryotic cell lines

Policy information about cell lines and Sex and Gender in Research

Cell line source(s)

Bone marrow (BM) cells were isolated from the femur and tibia of C57BL/6 mice(both female and male) and differentiated into mature macrophages. THP-1 cells (ATCC TIB-202 ™) Immortalized human lung microvascular ECs (iMVECs) were gifted by N. Mangalmurti. and described previously(Zhao et al. Science Advance. 2020).

Authentication

BM derived macrophages (BMDMs), which were validated by flow cytometry analysis (CD11b+F4/80+) , as reported previously(Toda G, et al. STAR Protoc. 2020. PMID: 33458708

Mycoplasma contamination

Cell lines were not tested for mycoplasma contamination

Commonly misidentified lines
(See ICLAC register)

These cell lines we used were not listed in commonly misidentified lines in ICLAC Register.

Animals and other research organisms

Policy information about studies involving animals; ARRIVE guidelines recommended for reporting animal research, and Sex and Gender in Research

Laboratory animals	C57BL6/J mice were bred in our own colony and were originally derived from Jackson Labs Stock #000664. All mice were Sparcl1flox (noted as Sparcl1flox/flox) or R26-LSL-Sparcl1 (noted as Sparcl1+/WT or Sparcl1+/+) mice were crossed with VECadCreERT2 (Cdh5CreERT2) mice 59 to produce VECadCreERT2; Sparcl1flox/flox mice , VECadCreERT2 ; Sparcl1+/WT , and VECadCreERT2 ; Sparcl1+/+ mice. Sparcl1flox/flox mice lacking Cre or VECadCreERT2; Sparcl1WT/WT mice were used as the conditional endothelial Sparcl1 knockout or overexpression control mice, respectively. Tlr4-/- mice (Jax. Stock#029015) were kindly gifted by Dr. Igor E. Brodsky. Control animals were co-housed with experimental groups .In this study, all mice were used at 6 to 8 weeks old, and mice of both sexes were used in equal proportions, and animals were housed in a specific pathogen-free colony, the number of mice used for individual in vivo experiments is specified in the figure legends. All animals euthanized using isoflurane in accordance with the University of Pennsylvania Animal Protocol (#806262).
Wild animals	No wild animal was involved in this study.
Reporting on sex	Both female and male mice were used in roughly equal proportions.
Field-collected samples	The study did not involve samples collected from the field.
Ethics oversight	All animal experiments were carried out under the guidelines set by the University of Pennsylvania's Institutional Animal Care and Use Committees, protocol #806262, and followed all National Institutes of Health (NIH) Office of Laboratory Animal Welfare regulations.

Note that full information on the approval of the study protocol must also be provided in the manuscript.

Clinical data

Policy information about clinical studies

All manuscripts should comply with the ICMJE guidelines for publication of clinical research and a completed CONSORT checklist must be included with all submissions.

Clinical trial registration	N/A
Study protocol	N/A
Data collection	N/A
Outcomes	N/A

Plants

Seed stocks	N/A
Novel plant genotypes	N/A
Authentication	N/A

Flow Cytometry

Plots

Confirm that:

- [x] The axis labels state the marker and fluorochrome used (e.g. CD4-FITC).
- [x] The axis scales are clearly visible. Include numbers along axes only for bottom left plot of group (a 'group' is an analysis of identical markers).
- [x] All plots are contour plots with outliers or pseudocolor plots.
- [x] A numerical value for number of cells or percentage (with statistics) is provided.

Methodology

Sample preparation	Details of sample preparation are provided in the Method section, including tissue-processing steps. Briefly, tissues samples were chemically disruption and filtered through a 70 μM strainer. Then the suspensions were incubated with red blood cell lysis buffer and washed with PBS or sort buffer. Single-cell suspensions were obtained and stained with antibodies according to the manufacturer's protocols, and then analyzed by flow cytometry.
Instrument	BD FACSAria Fusion Sorter and Symohony A3 Lite (BD Biosciences)
Software	FACS Diva and FlowJo V10
Cell population abundance	The absolute cells >500000 were analyzed for fluorescent intensity in the defined gate.
Gating strategy	Briefly, single cells were selected by SSC-A and SSC-H plots. Live cells were selected as defined by Live Dead Stain-negativity. Immune cells were gated by CD45+ cells. Total lung macrophages were gated by CD45+Ly6G-CD64+F4/80+ cells, alveolar macrophages were gated by CD45+Ly6G-CD64+F4/80+SiglecF+ cells, interstitial and recruited macrophages were gated by CD45+Ly6G-CD64+F4/80+SiglecF- cells, and then sub-gated M1-like (CD86+CD206-) and M2-like (CD86-CD206+) macrophages. Endothelial cells were gated by CD45-CD326-CD31+ cells. Epithelial cells were gated by CD45- CD326+ cells. B cells were gated by CD45+CD3-B220+cells. T cells were gated by CD45+CD3+B220- cells. ILC cells were gated by CD45+CD3-B220-Ly6G-CD11b-CD127+ cells, neutrophils were gated by CD11b+Ly6G+, inflammatory monocytes were gated by CD11b +Ly6C+. NK cells were gated by CD45+CD3-B220-Ly6G-CD11b-CD127-NK1.1+ cells. Detailed gating strategies were provided in the Supplementary Figures.

☒ Tick this box to confirm that a figure exemplifying the gating strategy is provided in the Supplementary Information.
